# Multilevel Models for Intensive Longitudinal Data with Heterogeneous Autoregressive Errors: The Effect of Misspecification and Correction with Cholesky Transformation

**DOI:** 10.3389/fpsyg.2017.00262

**Published:** 2017-02-24

**Authors:** Seungmin Jahng, Phillip K. Wood

**Affiliations:** ^1^Department of Psychology, Sungkyunkwan University Seoul, South Korea; ^2^Department of Psychological Sciences, University of Missouri Columbia, MO, USA

**Keywords:** multilevel model, intensive longitudinal data, heterogeneous autocorrelation, Cholesky transformation, misspecification

## Abstract

Intensive longitudinal studies, such as ecological momentary assessment studies using electronic diaries, are gaining popularity across many areas of psychology. Multilevel models (MLMs) are most widely used analytical tools for intensive longitudinal data (ILD). Although ILD often have individually distinct patterns of serial correlation of measures over time, inferences of the fixed effects, and random components in MLMs are made under the assumption that all variance and autocovariance components are homogenous across individuals. In the present study, we introduced a multilevel model with Cholesky transformation to model ILD with individually heterogeneous covariance structure. In addition, the performance of the transformation method and the effects of misspecification of heterogeneous covariance structure were investigated through a Monte Carlo simulation. We found that, if individually heterogeneous covariances are incorrectly assumed as homogenous independent or homogenous autoregressive, MLMs produce highly biased estimates of the variance of random intercepts and the standard errors of the fixed intercept and the fixed effect of a level 2 covariate when the average autocorrelation is high. For intensive longitudinal data with individual specific residual covariance, the suggested transformation method showed lower bias in those estimates than the misspecified models when the number of repeated observations within individuals is 50 or more.

## Introduction

Recent developments in data collection methods in the behavioral and social sciences, such as Ecological Momentary Assessment (EMA) (Stone and Shiffman, 1994; Hufford et al., 2001), enabled researchers in this area to gather data with many repeated measurements and to examine more detailed features of intra-individual variations over time. Such data that consist of repeated observations on a large number of occasions for many individuals with relatively short time intervals are called intensive longitudinal data (ILD: Walls and Schafer, 2006; Bolger and Laurenceau, 2013).^1^

Multilevel models (MLMs) have been widely used statistical tools for the analysis of both ILD and traditional longitudinal data that involves a small to moderate number of repeated observations (Walls et al., 2006; Schwartz and Stone, 2007; Nezlek, 2012). In a typical analysis of traditional longitudinal data using MLMs, within-person residual distributions are assumed to be identical across individuals, which means that all participants in the data have the same residual variance and autocorrelations. There may be several reasons for assuming homogenous within-person error structure in most applications of MLM with traditional longitudinal data. First, heterogeneous residual covariances may be less likely to exist after modeling random effects. Second, violation of the homogenous residual covariance assumption may not produce significant bias in estimation of the model parameters. Third, even if the heterogeneous residual covariances are likely to exist and need to be correctly specified, accurate estimation of individual covariance structure is not plausible with a small to moderate number of observations within individuals.

Due to the longitudinally intensive nature of assessments, however, the common practice of assuming homogenous residual covariance structure in MLM is questionable in case of ILD. First, heterogeneous residual covariances across individuals are very likely to exist in ILD. For example, many recent EMA studies have shown that there are substantial heterogeneity across individuals in the variance and autocorrelation of emotional states over time (e.g., Röcke et al., 2009; Kuppens et al., 2010; Hill and Updegraff, 2012; Koval and Kuppens, 2012; Tompson et al., 2012; Bresin, 2014; Ebner-Priemer et al., 2015). Second, little is known about the influence of violation of homogenous covariance structure on parameter estimation in MLMs with ILD. Misspecified error covariance structure in MLMs is known to produce inaccurate estimation in both fixed effects and variance components (Lange and Laird, 1989; Ferron et al., 2002, 2009; Kwok et al., 2007; Moeyaert et al., 2016) but the previous findings did not investigate the validity of common practice of assuming homogenous error structure in MLM with ILD when the within-person error structure is in fact different across individuals. Third, for such data, it is possible to reliably estimate individual-level covariance structure because a typical ILD has a sufficient number of observations within individuals.

In practice, researchers may fit a regression model with unstructured error covariance matrix to optimally estimate the fixed effects in ILD without modeling heterogeneous error covariance. Unstructured covariance structures in which every element is freely estimated from the data may represent a complicated correlational pattern among occasions. This approach, however, has several limitations when applied to ILD. First, multilevel analysis cannot be employed with unstructured residual covariance. Because unstructured covariance matrix is just identified, no additional random component can be modeled with it, that is random effects and their sources cannot be investigated. In addition, unstructured covariance matrix of ILD has too many parameters to be estimated, because the total number of parameters in the covariance matrix depends on the number of occasions, i.e., *n*(*n*+1)/2, where *n* is the number of occasions. For example, if the number of observations within each individual is 100, the number of parameters in the unstructured covariance is 5050. Models with a large number of parameters may suffer from non-convergence, under-identification, and/or non-positive definite solutions. This is especially true when the number of individuals is less than the number of occasions, which is often found in some ILD studies.

In short, heterogeneous variances and autocorrelations across individuals are likely to exist in ILD and, if ignored, may raise serious problems in estimating effects of covariates in MLM. However, relatively little is known about the influence of violation of homogenous covariance structure on parameter estimation in multilevel models with ILD. Use of unstructured error covariance structure does not successfully address the issue when researchers are interested in random effects or the number of measurement occasions is large.

Alternatively, a transformation method for regression with autocorrelated errors can be applied to the multilevel analysis of ILD in this context. A regression model for a single time-series with autocorrelated residuals can be correctly estimated by fitting OLS regression using data transformed by the inverse of Cholesky factor of the residual covariance matrix (Cochrane and Orcutt, 1949; Watson, 1955). The Cholesky transformation for a single time-series regression can be extended to a multiple time-series regression or MLM for ILD with heterogeneous autocorrelated errors. Specifically, MLM with Cholesky transformation method for ILD estimates a transformation matrix for each individual in the first step and then fit a multilevel model on the transformed data. By this transformation method, researchers can analyze multilevel models for ILD without assuming unrealistic homogenous autocorrelated residuals. In addition, this method does not require a greater number of individuals than the number of measurement occasions.

The present study has two aims. First, we introduce the Cholesky transformation method to model ILD with heterogeneous autoregressive error covariance structure. The transformation is designed to provide a legitimate application of MLM to a serially, and differently, correlated intensive longitudinal data. Second, the effects of misspecifying heterogeneous covariance structure as commonly used homogenous ones are investigated and compared to the result from the transformation method. Because the effect of the misspecification cannot be easily driven analytically and the suggested transformation method is expected to be valid asymptotically, we performed a Monte Carlo simulation by varying sample size, number of occasions, and average residual autocorrelation and then compared relative biases in estimation of fixed effects, random components, and the standard errors of fixed effects between misspecified models and the suggested method. In the next sections, a brief introduction to multilevel models is provided and issues in modeling residual covariance structure in MLMs are discussed. A multilevel modeling approach that uses transformation of an autocorrelated error structure into an independent structure will then be introduced, followed by the simulation study.

## Multilevel models with heterogeneous covariance structure

### Covariance structure in multilevel models for longitudinal data

In a longitudinal design, observations can be thought of as actualizations of a two-level data structure in which repeated observations (level-1) are nested within individuals (level-2). In a matrix form, the regression equation for individual *i* is expressed as


(1) $$y_{i}=X_{i}\gamma +Z_{i}u_{i}+e_{i},$$

where **X**_**i**_ = **Z**_**i**_**C**_**i**_, **Z**_**i**_ is the matrix of time-varying, within individual covariates including a column vector of ones, **C**_**i**_ is the matrix of individual-level covariates, **γ** is the vector of the fixed effects, and **u**_**i**_ is the vector of random effects.

A typical two-level linear model has two error terms, **u**_**i**_ and **e**_**i**_. The two error terms are assumed to be normally distributed with $E\left[\begin{array}{c} u_{i}\\ e_{i} \end{array}\right]=\left[\begin{array}{c} 0\\ 0 \end{array}\right]$ and $Cov\left[\begin{array}{c} u_{i}\\ e_{i} \end{array}\right]=\left[\begin{array}{cc} G_{i} & 0\\ 0 & R_{i} \end{array}\right]$. The mixture of the two normal distributions results in a multivariate normal distribution of **y**_**i**_, **y**_**i**_ ~ *N*(**X**_**i**_**γ**, **Σ**_**i**_), where $\Sigma_{i}=Z_{i}G_{i}Z_{i}^{\prime }+R_{i}$. For the entire observations $y=(y_{1}^{\prime },y_{2}^{\prime },\ldots ,y_{N}^{\prime }{)}^{\prime }$, the MLM is written as


(2) y=Xγ+Zu+e,

where $X=\left[\begin{array}{c} X_{1}\\ X_{2}\\ \vdots \\ X_{N} \end{array}\right]$, **Z** is the block diagonal matrix of **Z**_**i**_, i.e., $Z=\left[\begin{array}{cccc} Z_{1} & 0 & \cdots & 0\\ 0 & Z_{2} & \cdots & 0\\ \vdots & \vdots & \ddots & \vdots \\ 0 & 0 & \cdots & Z_{N} \end{array}\right],u=({u^{\prime }}_{1},{u^{\prime }}_{2},\ldots ,{u^{\prime }}_{N}{)}^{\prime }$, and $e=({e^{\prime }}_{1},{e^{\prime }}_{2},\ldots ,{e^{\prime }}_{N}{)}^{\prime }.$ The random vectors **u** and **e** are normally distributed with $E\left[\begin{array}{c} u\\ e \end{array}\right]=\left[\begin{array}{c} 0\\ 0 \end{array}\right]$ and $Cov\left[\begin{array}{c} u\\ e \end{array}\right]=\left[\begin{array}{cc} G & 0\\ 0 & R \end{array}\right]$, where **G** and **R** are the block diagonal matrices of **G**_**i**_ and **R**_**i**_, and **y** ~ *N*(**Xγ, Σ**), where **Σ** = **ZGZ**′ + **R** is the block diagonal matrices of **Σ**_**i**_. In practice, **G**_**i**_ and **R**_**i**_ are assumed homogenous across all level-2 individuals (i.e., **G**_**1**_ = **G**_**1**_ = … = **G**_**N**_ and **R**_**1**_ = **R**_**1**_ = … = **R**_**N**_) in most applications.

Common use of MLMs for longitudinal data, including linear growth models, assumes an unstructured **G**_**i**_ matrix that allows estimation of variances and covariances of all random effects (e.g., $G_{i}=\left[\begin{array}{c} \sigma_{u0}^{2}\\ \sigma_{u0u1}\;\sigma_{u1}^{2} \end{array}\right]$), and an independent and identical **R**_**i**_ matrix (**R**_**i**_ = $\sigma_{e}^{2}$ **I**: ID). However, it is likely that residuals of an MLM for ILD have serial correlations across time even after modeling the fixed and random effects. If this is the case, independence assumption in **R**_**i**_ is not appropriate and a suitable covariance structure that models autocorrelations between successive residuals should be specified in **R**_**i**_. For example, a covariance matrix generated by a first order autoregressive process can be used to model such a structure. If observations are measured at equally spaced time, the first order autoregressive covariance structure, AR(1), with four occasions, is modeled as


$$R_{i}=\sigma^{2}\left[\begin{array}{cccc} 1 & \rho & \rho^{2} & \rho^{3}\\ \rho & 1 & \rho & \rho^{2}\\ \rho^{2} & \rho & 1 & \rho \\ \rho^{3} & \rho^{2} & \rho & 1 \end{array}\right],$$

where ρ is the first order autoregressive parameter, i.e., autocorrelation between observations measured at time *t* and *t*-1.

Because **Σ** is determined by two covariance matrices **G** and **R** as well as a design matrix of random effect **Z**, misspecification of **R** may affect the estimation of **G**, or vice versa. The main diagonal elements of the square root of the covariance matrix of $\hat{\gamma },C={\left[X^{\prime }\Sigma^{-1}X\right]}^{-1}$, are the true standard errors of $\hat{\gamma }$. Thus, misspecification of **R** may also affect the estimation of the standard errors of fixed effects. These effects of misspecification of **R** on the estimation of **γ** and **G** has been studied by several researchers, in the context of linear growth models (Lange and Laird, 1989; Ferron et al., 2002; Jacqmin-Gadda et al., 2007; Kwok et al., 2007).

Ferron et al. (2002) found that misspecification of AR(1) residual covariance structure as ID structure in linear growth models results in overestimation of both $\sigma_{u0}^{2}$ and $\sigma_{u1}^{2}$ in **G** when ρ = 0.3 or 0.6, although bias in estimation of $\sigma_{u1}^{2}$ is much smaller than that of $\sigma_{u0}^{2}$. They also found that the coverage rate of 95% confidence interval for the slope was smaller than the true nominal value of 0.95 when the number of individuals is small (*N* = 30). Following the previous findings, Kwok et al. (2007) investigated the effect of misspecification in **R**_**i**_ for various covariance structures, such as ID, AR(1), first order autoregressive and first order moving average, and second banded Toeplitz structure, and found that underspecification produced minor overestimation in the standard errors of the intercept and the slope as well as noticeable overestimation variance estimates in **G**. Jacqmin-Gadda et al. (2007) showed that the estimation of **γ** under the normal ID assumption of **R**_**i**_ is robust to heteroscedastic residuals, unless the residual variance is a function of individual-level covariates, and non-normal residuals. When residuals are serially correlated, however, estimation of **γ** was biased: The coverage rates of 95% confidence intervals for intercept, slope, individual-level covariate, and the interaction of the last two were significantly smaller than the nominal value of 0.95.

### Regression with autocorrelated errors in a single time series

For data consisting of a single time series, the effect of autocorrelated errors on estimation of regression parameters is well known and estimation of regressions with autocorrelated residuals has long been of interest to statisticians (Cochrane and Orcutt, 1949; Watson, 1955; Chipman, 1979; Maeshiro, 1980; Park and Mitchell, 1980; Harvey, 1981; Koreisha and Fang, 2001). Although traditional OLS estimation for linear regression model assumes independence of observations, time series data usually violate this assumption. Consider the standard regression model:


(3) y=Xβ+e,

where **y** = (*y*_1_, *y*_2_, …, *y*_*n*_)′, *n* is the number of repeated measurements, **X** is a matrix of covariates, **β** is the corresponding regression parameter vector, and **e** = (*e*_1_, *e*_2_, …, *e*_*n*_)′ is a random residual vector with a covariance matrix $\Sigma =\sigma_{e}^{2}V_{e}$. If *e*_*t*_ is independent and has constant variance across *t*, i.e., $\Sigma =\sigma_{e}^{2}I$ or **V**_*e*_ = **I**, we can apply ordinary least squares to estimate β such that


(4) $$\hat{\beta }={\left(X^{\prime }X\right)}^{-1}X^{\prime }y,$$

and its covariance matrix is $\sigma_{e}^{2}{\left(X^{\prime }X\right)}^{-1}$, where the square root of each diagonal element is the standard error of estimation for the corresponding parameter in **β**. The OLS estimator $\hat{\beta }$ in this case is known as an unbiased and efficient estimator, or the best linear unbiased estimator (BLUE), in the sense that it has the smallest variance among all possible linear unbiased estimators.

If *e*_*t*_ is serially correlated, i.e., $\Sigma \neq \sigma_{e}^{2}I,$ however, $\hat{\beta }$ is no longer efficient. In such cases, generalized least squares (GLS) is used to estimate **β** such that


(5) $$\tilde{\beta }={\left(X^{\prime }\Sigma^{-1}X\right)}^{-1}X^{\prime }\Sigma^{-1}y.$$

Alternatively, a suitable transformation of **y** can also be used. In that case, multiplying Equation 3 by a transformation matrix **A**, such that $A\Sigma A^{\prime }=\sigma_{w}^{2}I$, gives


(6) Ay=AXβ+Ae=AXβ+w

where **w** is a white noise vector with covariance matrix $\sigma_{w}^{2}I.$ Equation 6 then can be expressed as


(7) $$y^{*}=X^{*}\beta +w,$$

where y^*^ = **Ay** and X^*^ = **AX**. Equations (6, 7) provides a valid OLS estimator of **β**,


(8) $${\hat{\beta }}_{w}={\left(X^{\prime }A^{\prime }AX\right)}^{-1}X^{\prime }A^{\prime }Ay={\left(X^{\prime }\Sigma^{-1}X\right)}^{-1}X^{\prime }\Sigma^{-1}y,$$

because $\sigma_{w}^{2}\Sigma^{-1}=A^{\prime }A$. The transformation matrix **A** is obtained as **A = L^−1^**, where **L** denotes the Cholesky root of **V**_*w*_, where ${\text{V}}_{w}={\left(\sigma_{w}^{2}\right)}^{-1}\Sigma$ (i.e., $\Sigma =\sigma_{w}^{2}{\text{V}}_{w}$), that is $V_{w}=LL^{\prime }$ with **L** lower triangular. If we know the covariance matrix **Σ** or **V**, Equations (5, 8) can directly be applied and the two methods will produce identical estimates of **β** (i.e., $\tilde{\beta }={\hat{\beta }}_{w}$). If not, the GLS estimation involves complicated estimation of **Σ** but the transformation method may use an alternative algorithm.

One possible approach for estimation of **Σ** (and thus a transformation matrix **A**) is to construct **Σ** from a known autocorrelation structure. Pioneering work in this approach was done by Cochrane and Orcutt (1949) for the simple Markov process. For a Markov process, *e*_*t*_ = ϕ*e*_*t*−1_ + *w*_*t*_, *w*_*t*_ ~ *N*(0, $\sigma_{w}^{2}$) or AR(1) process, autocovariance γ(*h*) is well known to be expressed as


(9) $$\gamma (h)=\frac{\sigma_{w}^{2}\rho^{h}}{1-\rho^{2}},$$

where ρ = ϕ is the first order autocorrelation (see Shumway and Stoffer, 2011, pp. 85–86). Autocovariance matrix **Σ** is then


(10) $$\Sigma =\frac{\sigma_{w}^{2}}{1-\rho^{2}}\left[\begin{array}{ccccc} 1 & \rho & \rho^{2} & \cdots & \rho^{n}\\ \rho & 1 & \rho & \cdots & \rho^{n-1}\\ \rho^{2} & \rho & 1 & \cdots & \rho^{n-2}\\ \vdots & \vdots & \vdots & \ddots & \vdots \\ \rho^{n} & \rho^{n-1} & \rho^{n-2} & \cdots & 1 \end{array}\right],$$

and its inverse matrix is


(11) $$\Sigma^{-1}=\frac{1}{\sigma_{w}^{2}}\left[\begin{array}{cccccc} 1 & -\rho & 0 & \cdots & 0 & 0\\ -\rho & 1+\rho^{2} & -\rho & \cdots & 0 & 0\\ 0 & -\rho & 1+\rho^{2} & \cdots & 0 & 0\\ \vdots & \vdots & \vdots & \ddots & \vdots & \vdots \\ 0 & 0 & 0 & \cdots & 1+\rho^{2} & -\rho \\ 0 & 0 & 0 & \cdots & -\rho & 1 \end{array}\right].$$

Assuming a simple Markov process, the GLS estimator $\tilde{\beta }$ can be obtained by Equation 5, where **Σ^−1^** is specified as in Equation 11.

To form a transformation matrix **A**, the inverse of Cholesky factor of **V**_*w*_ (i.e., the transpose of Cholesky factor of $V_{w}^{-1}$, where $V_{w}^{-1}=\sigma_{w}^{2}\Sigma^{-1}$) then can be obtained from **Σ**^**−1**^, given by


(12) $$L^{-1}=\left[\begin{array}{cccccc} \sqrt{1-\rho^{2}} & 0 & 0 & \cdots & 0 & 0\\ -\rho & 1 & 0 & \cdots & 0 & 0\\ 0 & -\rho & 1 & \cdots & 0 & 0\\ \vdots & \vdots & \vdots & \ddots & \vdots & \vdots \\ 0 & 0 & 0 & \cdots & 1 & 0\\ 0 & 0 & 0 & \cdots & -\rho & 1 \end{array}\right].$$

Using Equation 12, a valid OLS estimator ${\hat{\beta }}_{w}$ is obtained through Equation 8 (Judge et al., 1985). Cochrane and Orcutt (1949) did not provide the exact form of Equation 12 but a similar idea of transformation was offered. If **Σ**^**−1**^ is successfully estimated, either GLS estimation or OLS estimation with transformation can be used. For the general AR(*p*) model, *e*_*t*_ = ϕ_1_*e*_*t*−1_ + ϕ_2_*e*_*t*−2_ + … + ϕ_*p*_*e*_*t*−*p*_ + *w*_*t*_, however, estimation of **Σ** is challenging because the GLS approach requires a complicated nonlinear parameterization of γ(*h*) and **Σ**, and the calculations become more complicated as *p* increases. Because the lower triangular matrix **L**^−1^ is much simpler than **Σ** for the general AR(*p*) model, the transformation approach is exclusively used to correct higher order autoregressive error process for a single time series (SAS Institute Inc, 2013, p. 364).

### Correction for heterogeneous autocorrelations for ILD

Application of the transformation method to ILD is straightforward. With a number of repeated observations within individuals, transformation of each single series may correct autocorrelated errors estimated separately by each individual. A time series model for individual *i* can be written as


(13) $$y_{i}=Z_{i}\beta_{i}+e_{i},$$

where **Z**_**i**_ is time-varying covariates. Equation 13 can be extended to a multilevel format as


(14) $$y_{i}=X_{i}\gamma +Z_{i}u_{i}+e_{i},$$

where **X**_**i**_ = **Z**_**i**_**C**_**i**_, **Z**_**i**_ is the matrix of time-varying covariates, **C**_**i**_ is the matrix of individual-level covariates, **γ** is the fixed effect, and **u**_**i**_ is the random effect, respectively, as specified in Equation 1. The random effect **u**_**i**_ and the residual **e**_**i**_ are assumed to be normally distributed with $E\left[\begin{array}{c} u_{i}\\ e_{i} \end{array}\right]=\left[\begin{array}{c} 0\\ 0 \end{array}\right]$ and $Cov\left[\begin{array}{c} u_{i}\\ e_{i} \end{array}\right]=\left[\begin{array}{cc} G_{i} & 0\\ 0 & R_{i} \end{array}\right].$

If each individual has one's own covariance for **e**_**i**_, (i.e., **R**_**1**_ ≠ **R**_**1**_ ≠ … ≠ **R**_**N**_, for *i* = 1, 2, …, *N*), the transformed equation for each individual will be given by


(15) $$\begin{array}{l} A_{i}y_{i}=A_{i}X_{i}\gamma +A_{i}Z_{i}u_{i}+A_{i}e_{i}\\ \;=A_{i}X_{i}\gamma +A_{i}Z_{i}u_{i}+w_{i}, \end{array}$$

where random effect **u**_**i**_ and residual **w**_**i**_ are then normally distributed with $E\left[\begin{array}{c} u_{i}\\ w_{i} \end{array}\right]=\left[\begin{array}{c} 0\\ 0 \end{array}\right]$ and $Cov\left[\begin{array}{c} u_{i}\\ w_{i} \end{array}\right]=\left[\begin{array}{cc} G_{i} & 0\\ 0 & \sigma_{w_{i}}^{2}I_{i} \end{array}\right]$ and **A**_**i**_ is the inverse of Cholesky factor of ${\text{V}}_{\text{i}}={\left(\sigma_{w_{i}}^{2}\right)}^{-1}{\text{R}}_{\text{i}}$ (i.e., ${\text{R}}_{\text{i}}=\sigma_{w_{i}}^{2}{\text{V}}_{\text{i}}$). For the entire system, the transformed equation is written as


(16) $$\begin{array}{l} Ay=AX\gamma +AZu+Ae\\ \;=AX\gamma +AZu+w, \end{array}$$

where **A** is the block diagonal matrix of **A**_**i**_ and **X**, **Z**, and **u** are specified as in Equation 2. Assuming homogenous variance of the transformed residuals, the transformed variables **Ay**, **AZ**, and **AX** can be used in a multilevel model with the ID residual structure. Correction through Equation 16 is expected to reduce bias, if any, in estimation of the parameters when there are heterogeneous autocorrelations in the data.

The correction procedure of multilevel models with heterogeneous autocorrelations is summarized as

1. Fit Equation 13 by OLS estimation for each individual. Obtain ${\hat{y}}_{i}=Z_{i}{\hat{\beta }}_{i}$.
2. Calculate residuals as ${\hat{e}}_{i}=y_{i}-{\hat{y}}_{i}$ and investigate autocorrelations for ${\hat{e}}_{i}$
3. Define order *p* of AR(*p*) model for each individual by investigating autocorrelation patterns in residuals.
4. Apply transformation procedure for each individual and obtain the transformed data y^*^ = **Ay**, Z^*^ = **AZ**, and X^*^ = **AX**.
5. Fit the intended MLM using y^*^, Z^*^, and X^*^ assuming the ID or other independent residual structure.

Simply speaking, the above procedure consists of two steps. In the first step, transformed variables are obtained from a regression-with-autoregressive-error model for each individual.^2^ In the second step, the intended MLM is applied to the transformed variables obtained in the first step. We call this correction method as the multilevel model with Cholesky transformation (MLM-CT). The MLM-CT has several strengths in correction of autocorrelation in error structure for intensive longitudinal data. First, correction is applied for by each individual, without unrealistic identical distribution assumption in ILD. Second, time intervals between successive observations are not restricted to be equal across different individuals, although time intervals within individuals are restricted to be similarly spaced.^3^ Third, the correction is applicable to even higher order autoregressive error structure of general AR(*p*) models and, if required, allows different orders of autoregressive processes across individuals.

The suggested procedure may seem to be complicated to employ for applied researchers but can be easily done by using a commercial statistical software available. A SAS example code for the MLM-CT along with two commonly used multilevel models and the results of the analyses with a simulated data are presented in Appendix, which can be found in the Supplementary Material for this article.

## Performance in estimation of MLMs with heterogeneous autoregressive errors: a simulation study

The previous studies reported that misspecification of error covariance structure is associated with overestimation of variance components of the random effects and the standard errors of the fixed effects (Ferron et al., 2002; Kwok et al., 2007). We also focused on the effect of misspecification of **R** on the estimation of fixed effects and the variance components in **G**, in the sense that if MLMs assume homogenous **R**_**i**_, when it is in fact heterogeneous, the misspecified **R**_**i**_ may cause bias in estimation of the parameters in MLMs. To this end, a simulation study was conducted in which data generated from a longitudinal multilevel structure with heterogeneous autoregressive error process were analyzed by MLMs with homogenous assumption. The two most commonly used covariance structures for the analysis of longitudinal data, that is, ID and AR(1) were used to build misspecified models. In addition, the estimated parameters of the MLM with Cholesky transformation was evaluated against those of the misspecified models.

### Method

For simplicity, the following two-level linear mixed model was used to generate data:


(17) $$y_{ti}=\gamma_{00}+\gamma_{10}z_{ti}+\gamma_{01}c_{i}+\gamma_{11}c_{i}z_{ti}+u_{0i}+u_{1i}z_{ti}+e_{ti},$$

where γ_00_ is the fixed intercept, γ_10_ is the fixed effect of a time-varying covariate *z*_*ti*_, γ_01_ is the fixed effect of a time-invariant covariate *c*_*i*_, γ_11_ is the fixed effect of the cross-level interaction of *z*_*ti*_ and *c*_*i*_, *u*_0*i*_ is the random intercept, and *u*_1*i*_ is the random effect of *z*_*ti*_.^4^ The time-varying covariate *z*_*ti*_ and the time-invariant covariate *c*_*i*_ were generated from the standard normal distribution. Parameters of all fixed effects were set to 1. The random effect *u*_0*i*_ and *u*_1*i*_ were distributed multivariate normal as (u0iu1i)N~([00], [σu02σu0u1  σu12]), where $\sigma_{u0}^{2}=0.5$, $\sigma_{u1}^{2}=0.5$, and σ_*u*0*u*1_ = 0.15 (i.e., *r*_*u*0*u*1_ = .3). The errors were generated with a first order autoregressive model, *e*_*ti*_ = ρ_*i*_*e*_(*t*−1)*i*_ + *w*_*ti*_, *w*_*ti*_ ~ *N*(0, 1).

The autoregressive parameter ρ_*i*_ was allowed to vary across individuals, generated from a uniform distribution as ρ_*i*_ ~ *U*(ρ – 0.3, ρ + 0.3), where ρ = 0.0, 0.3, or 0.6, implying that *E*(ρ_*i*_) = 0.0, 0.3, or 0.6, respectively. Each data set was completely balanced with *L* (series length, or the number of observations within individuals) = 20, 50, 100, or 200 and *N* (the number of individuals) = 20, 50, 100, or 200. Accordingly, 4(*N*) × 4(*L*) × 3(ρ) = 48 conditions were obtained. In each condition, 500 data sets were simulated, resulting in a total of 24,000 data sets. Each data set was analyzed three times separately by three different MLMs: MLM with ID covariance structure (MLM-ID), MLM with a homogenous first order autoregressive covariance structure (MLM-AR), and MLM with Cholesky transformation (MLM-CT), resulting in a total of 72,000 analyses. For the transformation procedure in the first step of MLM-CT, a regression model with autoregressive errors was fitted for each individual and variables were transformed by using the AUTOREG procedure in SAS with ML estimation. After transformation, the transformed variables were fitted by the MLM with ID structure in the second step. All three MLMs were properly modeled and fitted using the MIXED procedure in SAS with restricted maximum likelihood estimation.

Bias in parameter estimation was investigated in terms of relative bias for the fixed effects γ_00_, γ_10_, γ_01_, and γ_11_ and the variance components, $\sigma_{u0}^{2}$, $\sigma_{u1}^{2}$, and $\sigma_{u0u1}$. Relative bias was calculated in percentage as $\frac{1}{R}\overset{R}{\underset{r=1}{\sum }}\frac{{\hat{\theta }}_{r}-\theta }{\theta }\times 100$, where θ is the true parameter value, ${\hat{\theta }}_{r}$ is the corresponding sample estimate of the *r*th sample, and *R* is the number of replications converged in each condition.^5^ Biases in the estimated standard error were also investigated. Relative bias of standard error for the fixed effects was also calculated in percentage as $\frac{1}{R}\overset{R}{\underset{r=1}{\sum }}\frac{{\hat{\theta }}_{r}-\theta }{\theta }\times 100$, where θ is the true standard error and ${\hat{\theta }}_{r}$ is the estimated standard error for the *r*th sample.

The estimates of the fixed effects obtained by the two misspecified MLMs (i.e., MLM-ID and MLM-AR) were expected to be unbiased because misspecified error covariance structure is unlikely to influence bias in point estimation of the fixed effects. Estimates of the variance components of random effects, however, are likely to be biased for the two MLMs with misspecified covariance structures, especially for the variance of random intercept with high serial correlations (see Ferron et al., 2002; Kwok et al., 2007). This bias was expected to be greater for MLM-ID than MLM-AR because the first is more restricted by the independence assumption. In addition, MLM-CT is expected to reduce biases of estimates of the variance components in some conditions, but not in other conditions. Specifically, because a successful correction of the MLM-CT depends on valid transformation in the first step, which requires enough number of observations for each individual, a less biased estimation in the second step was expected not for the data with a relatively small to moderate number of repeated observations (e.g., *L* = 20 or 50) but for the data with a large number of observations (e.g., *L* = 100 or 200) for each individual. Bias in estimation of the standard error of estimation for the fixed effects is also more likely in the MLM-ID and the MLM-AR, for the data with a large number of repeated observations, than in the MLM-CT, because the estimated standard error is a function of the estimated variance components.

### Results

#### Bias in fixed effects

A total of 72,000 analyses were all converged. Relative biases for the estimates of the fixed effects (i.e., γ_00_, γ_10_, γ_01_, and γ_11_) are presented in Table 1. No significant bias was found in the estimation of the fixed effects across all the three methods.^6^ The result suggests that the estimates of the fixed effects obtained by MLMs with homogenous covariance assumption are not biased when the error covariance structure is in fact heterogeneous. This is true whether the sample size is small or large, series length is short or long, and the average error autocorrelation is null or high. The result also showed that the transformation procedure does not produce biased estimates for the fixed effects in MLMs.

**Table 1:** **Relative Bias (%) of ${\hat{\gamma }}_{00}$, ${\hat{\gamma }}_{10}$, ${\hat{\gamma }}_{01}$ and ${\hat{\gamma }}_{11}$ under heterogeneous autocorrelations in error for the three different MLMs**.

***N***	***L***	***ρ* = 0.0**			***ρ* = 0.3**			***ρ* = 0.6**		
		**ID**	**AR(1)**	**CT**	**ID**	**AR(1)**	**CT**	**ID**	**AR(1)**	**CT**
** ${\hat{\gamma }}_{00}$ **										
20	20	−0.3	−0.3	−0.3	0.8	0.8	0.8	−0.4	−0.3	−0.5
	50	−0.2	−0.2	−0.2	1.1	1.1	1.0	−0.2	−0.2	0.0
	100	−0.8	−0.8	−0.8	0.9	0.9	0.9	0.2	0.2	0.0
	200	0.0	0.0	0.0	0.6	0.6	0.6	0.4	0.4	0.3
50	20	−0.4	−0.4	−0.3	−0.1	−0.1	−0.1	0.5	0.4	0.7
	50	0.2	0.2	0.2	0.4	0.4	0.4	0.3	0.3	0.4
	100	0.5	0.5	0.5	0.8	0.8	0.8	−0.7	−0.7	−0.8
	200	0.0	0.0	0.0	−0.6	−0.6	−0.6	−0.7	−0.7	−0.6
100	20	0.2	0.2	0.2	0.0	0.0	0.0	−0.2	−0.2	−0.1
	50	−0.7	−0.7	−0.7	−0.3	−0.3	−0.3	−0.3	−0.3	−0.2
	100	−0.5	−0.5	−0.5	−0.5	−0.5	−0.5	0.8	0.8	0.8
	200	−0.6	−0.6	−0.6	0.2	0.2	0.2	0.5	0.5	0.5
200	20	−0.7	−0.7	−0.7	0.2	0.2	0.2	0.4	0.4	0.3
	50	0.2	0.2	0.2	0.1	0.1	0.1	0.4	0.4	0.4
	100	−0.1	−0.1	−0.1	−0.1	−0.1	−0.1	0.0	0.0	0.0
	200	0.0	0.0	0.0	−0.1	−0.1	−0.1	0.1	0.1	0.1
** ${\hat{\gamma }}_{10}$ **										
20	20	−0.6	−0.6	−0.6	0.5	0.5	0.4	−1.1	−0.9	−1.0
	50	−0.5	−0.5	−0.5	0.4	0.4	0.4	−0.3	−0.2	−0.2
	100	−0.6	−0.6	−0.6	0.1	0.2	0.2	−0.6	−0.6	−0.6
	200	−0.8	−0.8	−0.8	−0.6	−0.6	−0.6	0.8	0.8	0.8
50	20	−0.4	−0.4	−0.4	−0.1	−0.1	−0.1	−0.2	−0.2	−0.2
	50	−0.2	−0.2	−0.2	0.5	0.5	0.6	1.0	1.0	1.0
	100	−0.2	−0.2	−0.2	1.1	1.1	1.1	0.3	0.2	0.2
	200	0.0	0.0	0.0	−0.5	−0.5	−0.5	0.0	0.0	0.0
100	20	0.5	0.5	0.6	0.0	−0.1	−0.1	−0.3	−0.2	−0.2
	50	−0.5	−0.5	−0.5	−0.5	−0.5	−0.4	0.6	0.6	0.6
	100	0.0	0.0	0.0	−0.5	−0.5	−0.5	−0.1	−0.2	−0.2
	200	−0.1	−0.1	−0.1	0.3	0.3	0.3	−0.1	−0.1	−0.1
200	20	−0.5	−0.5	−0.4	0.2	0.2	0.2	0.2	0.2	0.2
	50	−0.1	−0.1	−0.1	0.0	0.0	0.0	0.0	0.0	0.0
	100	0.1	0.1	0.1	0.0	0.0	−0.1	0.1	0.0	0.0
	200	0.3	0.3	0.3	−0.3	−0.3	−0.3	0.3	0.2	0.3
** ${\hat{\gamma }}_{01}$ **										
20	20	0.4	0.4	0.4	−0.2	−0.2	−0.2	0.5	0.4	0.6
	50	0.3	0.3	0.2	−1.1	−1.1	−1.2	1.1	1.2	1.4
	100	−1.3	−1.3	−1.3	−0.1	−0.1	−0.1	−0.2	−0.2	−0.4
	200	−1.1	−1.1	−1.2	−0.5	−0.5	−0.5	−1.5	−1.5	−1.6
50	20	−1.0	−1.0	−1.0	0.3	0.3	0.3	0.1	0.1	0.0
	50	0.4	0.4	0.4	0.1	0.1	0.1	−0.7	−0.7	−0.8
	100	−1.2	−1.2	−1.2	0.2	0.2	0.2	0.5	0.5	0.4
	200	0.2	0.2	0.2	−0.4	−0.4	−0.4	0.2	0.2	0.2
100	20	−0.1	−0.1	−0.2	−0.1	−0.1	0.0	0.3	0.2	0.2
	50	0.2	0.2	0.2	0.1	0.1	0.0	−0.1	−0.1	−0.2
	100	−0.1	−0.1	−0.1	0.2	0.2	0.2	0.0	−0.1	0.0
	200	0.3	0.3	0.3	−0.1	−0.1	−0.2	−0.5	−0.5	−0.5
200	20	−0.1	−0.1	0.0	−0.4	−0.4	−0.4	0.4	0.4	0.4
	50	0.1	0.1	0.1	0.0	0.0	0.0	−0.3	−0.3	−0.4
	100	−0.1	−0.1	−0.1	0.1	0.1	0.1	−0.3	−0.3	−0.3
	200	0.1	0.1	0.1	0.3	0.3	0.3	−0.1	−0.1	−0.1
** ${\hat{\gamma }}_{11}$ **										
20	20	0.8	0.8	0.7	−1.3	−1.3	−1.3	0.8	0.6	0.6
	50	1.0	1.0	1.0	−0.1	−0.1	0.0	0.7	0.6	0.6
	100	−0.3	−0.3	−0.3	0.1	0.1	0.1	−0.2	−0.2	−0.2
	200	−1.2	−1.1	−1.1	−0.1	−0.1	−0.1	−0.7	−0.8	−0.7
50	20	0.2	0.2	0.2	0.4	0.3	0.4	−1.3	−1.1	−1.1
	50	0.7	0.7	0.7	0.5	0.6	0.6	−0.5	−0.5	−0.5
	100	−0.4	−0.4	−0.3	0.6	0.6	0.6	0.6	0.5	0.5
	200	−0.7	−0.7	−0.7	−0.3	−0.3	−0.3	0.6	0.6	0.6
100	20	0.2	0.2	0.2	−0.1	−0.1	−0.1	−0.1	−0.2	−0.2
	50	−0.2	−0.2	−0.2	0.2	0.1	0.1	0.0	0.1	0.0
	100	0.0	0.0	0.0	−0.1	−0.1	−0.1	−0.2	−0.1	−0.1
	200	−0.3	−0.3	−0.3	−0.1	−0.1	−0.1	−0.3	−0.2	−0.3
200	20	0.1	0.1	0.1	−0.3	−0.2	−0.2	0.6	0.6	0.6
	50	0.4	0.4	0.4	0.1	0.1	0.1	−0.1	−0.1	−0.1
	100	−0.4	−0.4	−0.4	0.1	0.1	0.1	−0.3	−0.2	−0.2
	200	0.3	0.3	0.3	−0.1	−0.1	−0.1	−0.2	−0.2	−0.2

*ID, MLM with homogenous variance assumption; AR(1), MLM with homogenous first order autoregressive error structure; CT, MLM with Cholesky transformation. ρ, average autocorrelation; N, sample size; L, series length*.

#### Bias in variance and covariance of random effects

**Figure 1:**
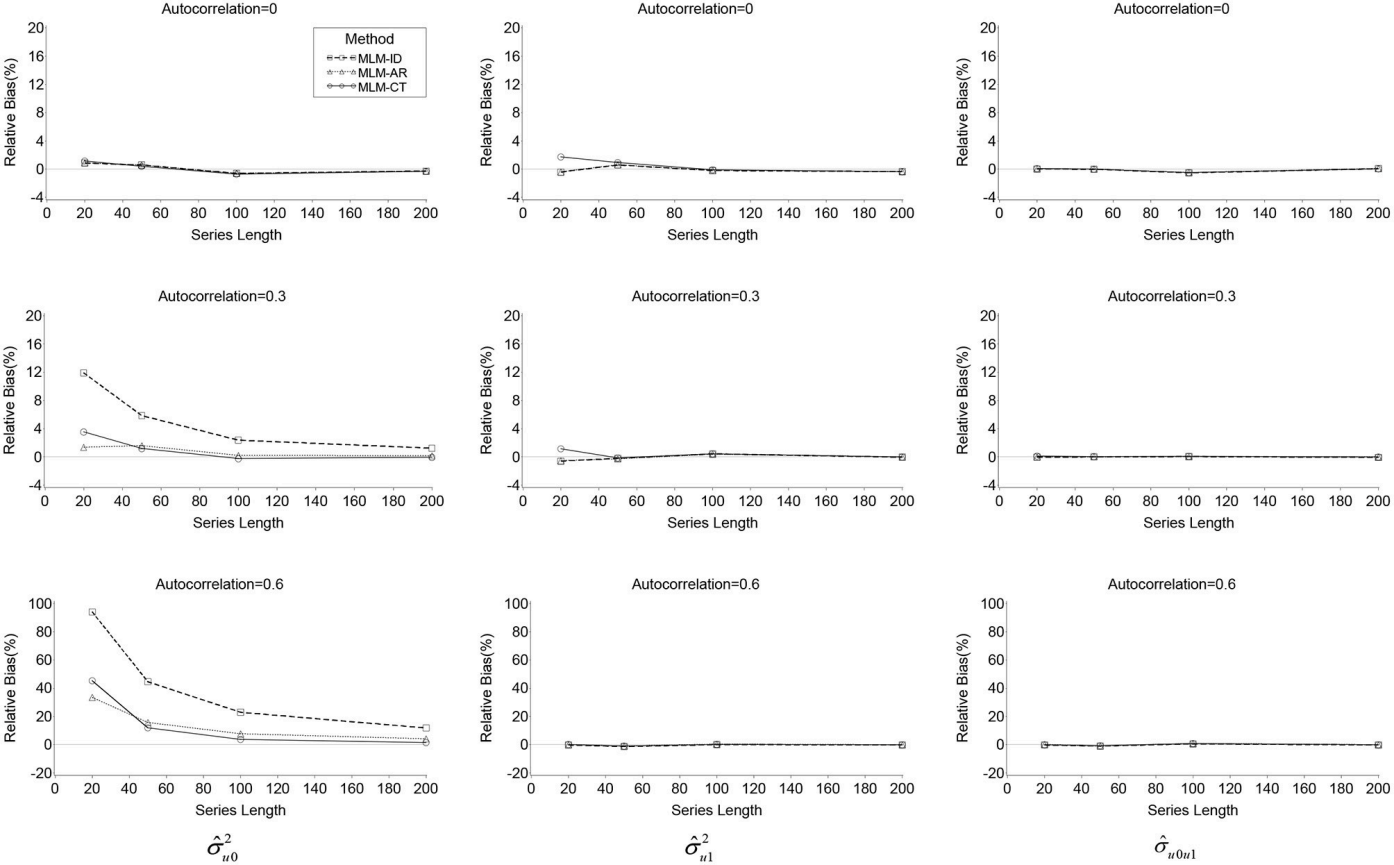
**Line plots of relative bias of ${\hat{\sigma }}_{u0}^{2}$, ${\hat{\sigma }}_{u1}^{2}$, and ${\hat{\sigma }}_{u0u1}$ produced by the three MLMs across autocorrelations and series lengths**.

Relative biases for the variance of the random effects ($\sigma_{u0}^{2}$ and $\sigma_{u1}^{2}$) and their covariance ($\sigma_{u0u1}$) are presented in Table 2. There was a positive bias in the estimation of $\sigma_{u0}^{2}$ for the two misspecified models, overall relative biases were 16.3% for the MLM-ID and 5.4% for the MLM-AR. For the MLM-ID, bias appeared particularly large when high autocorrelation was present (η^2^ = 0.24) or series length was short (η^2^ = 0.09) and even larger when both conditions occurred (η^2^ = 0.12) (see Table 2 and Figure 1). A similar pattern of significance of the effects was also found for the MLM-AR although the magnitude of the effects was smaller than that for the ID model: η^2^ = 0.02 for series length, η^2^ = 0.05 for autocorrelation, and η^2^ = 0.03 for their interaction. Bias was much higher in the MLM-ID than the MLM-AR (η^2^ = 0.26) when analyzed by 2(method) × 3(*N*) × 4(*L*) × 3(ρ) repeated ANOVA. This difference was greater especially when series length was short (η^2^ = 0.14), or autocorrelation was high (η^2^ = 0.33), and even greater when both conditions occurred (η^2^ = 0.17) (see Figure 1).

**Table 2:** **Relative bias (%) of ${\hat{\sigma }}_{u0}^{2}$, ${\hat{\sigma }}_{u1}^{2}$, and ${\hat{\sigma }}_{u0u1}$ under heterogeneous autocorrelations in error for the three different MLMs**.

***N***	***L***	***ρ* = 0.0**			***ρ* = 0.3**			***ρ* = 0.6**		
		**ID**	**AR(1)**	**CT**	**ID**	**AR(1)**	**CT**	**ID**	**AR(1)**	**CT**
** ${\hat{\sigma }}_{u0}^{2}$ **										
20	20	1.6	1.5	2.0	10.1	−0.8	0.5	98.3	35.9	49.7
	50	−0.8	−0.8	−1.0	6.6	2.4	2.3	45.5	14.6	12.0
	100	−0.6	−0.6	−0.8	1.3	−0.9	−1.4	22.5	7.1	3.4
	200	−0.8	−0.8	−0.9	1.4	0.3	0.2	11.9	4.2	1.9
50	20	0.7	0.8	0.9	11.7	1.2	4.0	93.0	32.5	44.9
	50	1.3	1.3	1.1	6.8	2.5	2.0	45.2	16.9	13.4
	100	−2.1	−2.1	−2.2	2.8	0.6	0.2	21.4	6.2	2.3
	200	−0.9	−0.9	−1.0	1.1	0.1	−0.1	11.0	3.0	0.6
100	20	0.8	0.8	1.0	12.9	2.6	4.7	93.5	33.4	44.3
	50	1.4	1.4	1.2	4.7	0.4	−0.1	44.4	15.8	11.4
	100	0.0	0.0	−0.1	3.2	1.0	0.6	24.0	8.8	4.0
	200	0.4	0.4	0.3	0.5	−0.6	−0.9	12.1	4.3	1.3
200	20	0.3	0.4	0.6	12.9	2.5	4.9	91.6	31.8	42.6
	50	0.4	0.4	0.2	5.3	1.0	0.5	43.4	15.0	10.7
	100	0.3	0.3	0.1	2.1	0.0	−0.4	24.1	8.9	4.7
	200	0.2	0.2	0.1	1.9	0.8	0.6	12.4	4.6	1.5
** ${\hat{\sigma }}_{u1}^{2}$ **										
20	20	−0.2	−0.2	1.9	−1.4	−1.0	0.8	0.2	0.1	0.5
	50	2.4	2.4	2.7	−1.9	−2.0	−1.9	−2.8	−2.5	−2.6
	100	−0.6	−0.6	−0.4	2.3	2.2	2.2	2.7	3.0	2.9
	200	−1.7	−1.7	−1.7	1.1	1.1	1.1	−0.4	−0.5	−0.5
50	20	−0.7	−0.7	1.6	−1.2	−1.3	0.4	−1.1	−1.3	−0.8
	50	0.2	0.2	0.4	−0.4	−0.5	−0.4	−1.3	−0.9	−1.0
	100	0.0	0.0	0.1	−0.9	−1.0	−0.9	−1.4	−1.1	−1.1
	200	−0.4	−0.4	−0.4	−0.4	−0.4	−0.4	0.8	0.8	0.7
100	20	0.0	0.0	1.9	0.2	0.1	1.8	−0.1	0.0	0.2
	50	−0.4	−0.4	−0.1	0.4	0.2	0.5	−0.5	−0.5	−0.5
	100	−0.3	−0.3	−0.2	0.1	0.1	0.1	−0.7	−0.5	−0.6
	200	1.0	0.9	1.0	−0.3	−0.3	−0.3	−0.5	−0.6	−0.6
200	20	−0.8	−0.8	1.4	0.1	0.1	1.7	0.3	0.2	0.6
	50	0.2	0.2	0.5	1.0	1.0	1.2	−0.5	−0.3	−0.4
	100	0.0	0.0	0.1	0.3	0.2	0.3	−0.5	−0.5	−0.6
	200	−0.3	−0.3	−0.3	−0.5	−0.5	−0.5	−0.6	−0.4	−0.5
** ${\hat{\sigma }}_{u0u1}$ **										
20	20	1.4	1.3	1.5	−0.5	−0.5	−0.3	0.5	0.7	0.3
	50	−0.5	−0.5	−0.5	−1.2	−1.3	−1.2	−3.7	−3.7	−3.6
	100	−2.3	−2.3	−2.3	1.2	1.1	1.2	3.1	3.2	3.2
	200	0.1	0.1	0.0	−0.9	−0.9	−0.9	0.2	0.1	0.1
50	20	−0.8	−0.8	−0.7	0.0	0.1	0.3	0.1	0.5	0.6
	50	−0.4	−0.4	−0.4	0.8	0.8	0.8	0.8	0.6	0.7
	100	−0.5	−0.5	−0.5	−0.8	−0.8	−0.8	−0.7	−0.5	−0.6
	200	−0.4	−0.4	−0.4	0.5	0.5	0.5	−0.4	−0.3	−0.2
100	20	−0.1	−0.1	−0.1	0.3	0.3	0.3	−0.8	−0.7	−1.1
	50	0.2	0.2	0.2	0.3	0.2	0.2	−0.3	−0.4	−0.2
	100	0.7	0.7	0.7	0.0	0.0	0.0	−0.4	−0.4	−0.3
	200	0.5	0.5	0.5	0.3	0.3	0.3	−0.5	−0.5	−0.5
200	20	−0.3	−0.3	−0.3	0.0	0.0	0.1	−0.6	−0.6	−0.7
	50	0.6	0.6	0.6	0.2	0.2	0.2	−0.5	−0.3	−0.2
	100	0.1	0.1	0.1	−0.2	−0.2	−0.2	0.4	0.4	0.4
	200	0.0	0.0	0.0	−0.1	−0.1	−0.1	−0.1	−0.1	0.0

*ID, MLM with homogenous variance assumption; AR(1), MLM with homogenous first order autoregressive error structure; CT, MLM with Cholesky transformation. ρ, average autocorrelation; N, sample size; L, series length*.

On the other hand, the transformation method did not completely eliminate bias in the estimates of $\sigma_{u0}^{2}$. The MLM-CT method also showed a significant relative bias (overall *RB* = 5.6%), especially when series length was short (η^2^ = 0.05), autocorrelation was high (η^2^ = 0.06), or both conditions occurred (η^2^ = 0.07). In fact, when series length was 20, regardless of the size of autocorrelation, the MLM-CT showed larger bias than the MLM-AR. However, when series length was 50 or more, the differences were indiscernible and, for ρ = 0.6, the MLM-CT showed smaller bias than the MLM-AR (see Figure 1).

By contrast, no significant bias was found in estimation of the variance of the random regression effect ($\sigma_{u1}^{2}$) and the covariance of the two random effects ($\sigma_{u0u1}$) for the three models. There was no considerable difference of the relative bias among the three models.

#### Bias in the standard error of estimation for fixed effects

Because of the bias in the estimated variance of the random intercept, the standard errors of estimation associated with the fixed effects are also expected to be biased. Table 3 presents relative bias of the estimated standard error for ${\hat{\gamma }}_{00}$, ${\hat{\gamma }}_{10}$, ${\hat{\gamma }}_{01}$, and ${\hat{\gamma }}_{11}$. Notice the great similarities of the results for the standard errors of ${\hat{\gamma }}_{00}$ and ${\hat{\gamma }}_{01}$ as well as for those of ${\hat{\gamma }}_{10}$ and ${\hat{\gamma }}_{11}$ in Table 3.

**Table 3:** **Relative bias (%) of the Standard Error of ${\hat{\gamma }}_{00}$, ${\hat{\gamma }}_{10}$, ${\hat{\gamma }}_{01}$ and ${\hat{\gamma }}_{11}$ under heterogeneous autocorrelations in error for the three different MLMs**.

***N***	***L***	***ρ* = 0.0**			***ρ* = 0.3**			***ρ* = 0.6**		
		**ID**	**AR(1)**	**CT**	**ID**	**AR(1)**	**CT**	**ID**	**AR(1)**	**CT**
***SE*** **(${\hat{\gamma }}_{00}$)**										
20	20	−0.8	−0.8	−0.6	−1.9	−2.1	−2.5	10.0	8.7	5.3
	50	−2.0	−2.0	−1.9	−0.6	−0.6	−0.5	4.3	3.5	0.0
	100	−1.8	−1.8	−1.8	−2.0	−2.0	−2.1	0.7	0.5	−1.3
	200	−1.8	−1.8	−1.8	−1.4	−1.4	−1.3	−0.4	−0.5	−1.0
50	20	−0.4	−0.4	−0.3	−0.4	−0.6	−0.2	10.6	9.1	5.9
	50	−0.1	−0.1	−0.1	0.3	0.3	0.2	5.5	5.0	1.9
	100	−1.6	−1.6	−1.6	−0.4	−0.4	−0.5	1.3	1.2	−0.6
	200	−1.0	−1.0	−1.0	−0.6	−0.6	−0.6	0.1	0.0	−0.6
100	20	−0.1	−0.1	0.0	0.4	0.3	0.5	11.2	9.6	6.3
	50	0.3	0.3	0.3	−0.4	−0.4	−0.5	5.6	5.0	1.6
	100	−0.3	−0.3	−0.3	0.1	0.0	0.0	2.8	2.5	0.5
	200	−0.1	−0.1	−0.1	−0.7	−0.7	−0.7	0.9	0.8	0.0
200	20	−0.2	−0.2	0.0	0.6	0.4	0.7	10.8	9.4	5.9
	50	0.0	0.0	0.0	0.0	0.0	0.0	5.5	4.9	1.5
	100	0.0	0.0	−0.1	−0.3	−0.4	−0.4	2.9	2.7	0.9
	200	−0.1	−0.1	−0.1	0.2	0.2	0.2	1.1	1.0	0.3
***SE*** **(${\hat{\gamma }}_{10}$)**										
20	20	−1.2	−1.3	−0.9	−1.1	−1.8	−1.4	2.3	−1.4	−1.1
	50	−0.2	−0.2	−0.3	−1.8	−2.3	−2.3	−0.8	−2.7	−2.7
	100	−1.7	−1.7	−1.7	0.0	−0.3	−0.3	1.0	0.1	0.1
	200	−2.1	−2.1	−2.2	−0.6	−0.7	−0.7	−0.9	−1.5	−1.6
50	20	−0.6	−0.6	−0.3	0.0	−0.9	−0.5	2.7	−1.1	−0.8
	50	−0.3	−0.3	−0.4	−0.3	−0.7	−0.7	0.8	−1.0	−1.0
	100	−0.4	−0.4	−0.5	−0.8	−1.0	−1.0	−0.1	−1.1	−1.1
	200	−0.7	−0.7	−0.7	−0.6	−0.7	−0.7	0.4	−0.2	−0.2
100	20	0.0	0.0	0.2	0.8	−0.1	0.3	3.4	−0.2	0.0
	50	−0.3	−0.3	−0.4	0.4	−0.1	−0.1	1.5	−0.5	−0.5
	100	−0.3	−0.3	−0.4	0.1	−0.2	−0.2	0.5	−0.5	−0.5
	200	0.3	0.3	0.2	−0.3	−0.4	−0.4	0.1	−0.5	−0.5
200	20	−0.2	−0.2	0.1	0.9	0.1	0.4	3.8	0.0	0.2
	50	0.1	0.1	0.1	0.8	0.4	0.4	1.6	−0.3	−0.3
	100	−0.1	−0.1	−0.1	0.2	0.0	0.0	0.7	−0.4	−0.4
	200	−0.2	−0.2	−0.3	−0.2	−0.3	−0.3	0.2	−0.3	−0.4
***SE*** **(${\hat{\gamma }}_{01}$)**										
20	20	−0.8	−0.9	−0.6	−1.8	−2.0	−2.4	10.1	8.8	5.4
	50	−2.0	−2.0	−2.0	−0.6	−0.6	−0.6	4.7	3.9	0.1
	100	−1.8	−1.8	−1.8	−2.0	−2.0	−2.1	0.6	0.4	−1.2
	200	−1.8	−1.8	−1.8	−1.3	−1.4	−1.3	−0.4	−0.5	−1.1
50	20	−0.4	−0.4	−0.3	−0.4	−0.5	−0.2	10.5	9.1	5.8
	50	−0.1	−0.1	−0.1	0.3	0.3	0.2	5.3	4.8	1.8
	100	−1.6	−1.6	−1.6	−0.4	−0.4	−0.4	1.2	1.1	−0.6
	200	−1.0	−1.0	−1.0	−0.6	−0.6	−0.6	0.0	−0.1	−0.6
100	20	−0.1	−0.1	0.0	0.4	0.2	0.4	11.2	9.7	6.2
	50	0.3	0.3	0.3	−0.3	−0.4	−0.5	5.5	4.9	1.6
	100	−0.3	−0.3	−0.3	0.0	0.0	0.0	2.6	2.4	0.5
	200	−0.1	−0.1	−0.1	−0.7	−0.7	−0.7	0.9	0.8	0.0
200	20	−0.2	−0.2	0.0	0.6	0.5	0.8	10.7	9.2	5.9
	50	0.0	0.0	0.0	0.0	0.0	0.0	5.5	4.9	1.6
	100	−0.1	−0.1	−0.1	−0.3	−0.4	−0.4	2.9	2.7	0.9
	200	−0.1	−0.1	−0.1	0.2	0.2	0.2	1.1	1.1	0.3
***SE*** **(${\hat{\gamma }}_{11}$)**										
20	20	−1.2	−1.3	−0.9	−1.1	−1.8	−1.4	2.4	−1.4	−1.2
	50	−0.2	−0.2	−0.3	−1.8	−2.3	−2.3	−0.8	−2.7	−2.7
	100	−1.7	−1.7	−1.7	0.0	−0.3	−0.3	1.0	0.1	0.1
	200	−2.1	−2.1	−2.2	−0.6	−0.7	−0.7	−0.9	−1.5	−1.6
50	20	−0.6	−0.6	−0.3	0.0	−0.9	−0.5	2.7	−1.1	−0.8
	50	−0.3	−0.3	−0.4	−0.3	−0.7	−0.7	0.8	−1.0	−1.0
	100	−0.4	−0.4	−0.5	−0.8	−1.0	−1.0	−0.1	−1.1	−1.1
	200	−0.7	−0.7	−0.7	−0.6	−0.7	−0.7	0.4	−0.2	−0.2
100	20	0.0	0.0	0.2	0.8	−0.1	0.3	3.4	−0.2	0.0
	50	−0.3	−0.3	−0.4	0.4	−0.1	−0.1	1.5	−0.5	−0.5
	100	−0.3	−0.3	−0.4	0.1	−0.2	−0.2	0.5	−0.5	−0.5
	200	0.3	0.3	0.2	−0.3	−0.4	−0.4	0.1	−0.5	−0.5
200	20	−0.2	−0.2	0.1	0.9	0.1	0.4	3.8	0.0	0.2
	50	0.1	0.1	0.1	0.8	0.4	0.4	1.6	−0.3	−0.3
	100	−0.1	−0.1	−0.1	0.2	0.0	0.0	0.7	−0.4	−0.4
	200	−0.2	−0.2	−0.3	−0.2	−0.3	−0.3	0.2	−0.3	−0.4

*ID, MLM with homogenous variance assumption; AR(1), MLM with homogenous first order autoregressive error structure; CT, MLM with Cholesky transformation; SE, standard error. ρ, average autocorrelation; N, sample size; L, series length*.

Overall relative bias of the estimated standard error of the estimates for the fixed intercept (${\hat{\gamma }}_{00}$) was found in the ID model (*RB* = 1.2%) and the AR model (*RB* = 0.9%) but not for the MLM-CT model (*RB* = 0.2%). Bias of the ID model was mainly affected by series length (η^2^ = 0.01), autocorrelation (η^2^ = 0.04), and the interaction of the two (η^2^ = 0.02). Bias of the AR model was also affected by series length (η^2^ = 0.01), autocorrelation (η^2^ = 0.03), and the interaction of the two (η^2^ = 0.02). Bias of the MLM-CT model was affected by the interaction of series length by autocorrelation (η^2^ = 0.01).

**Figure 2:**
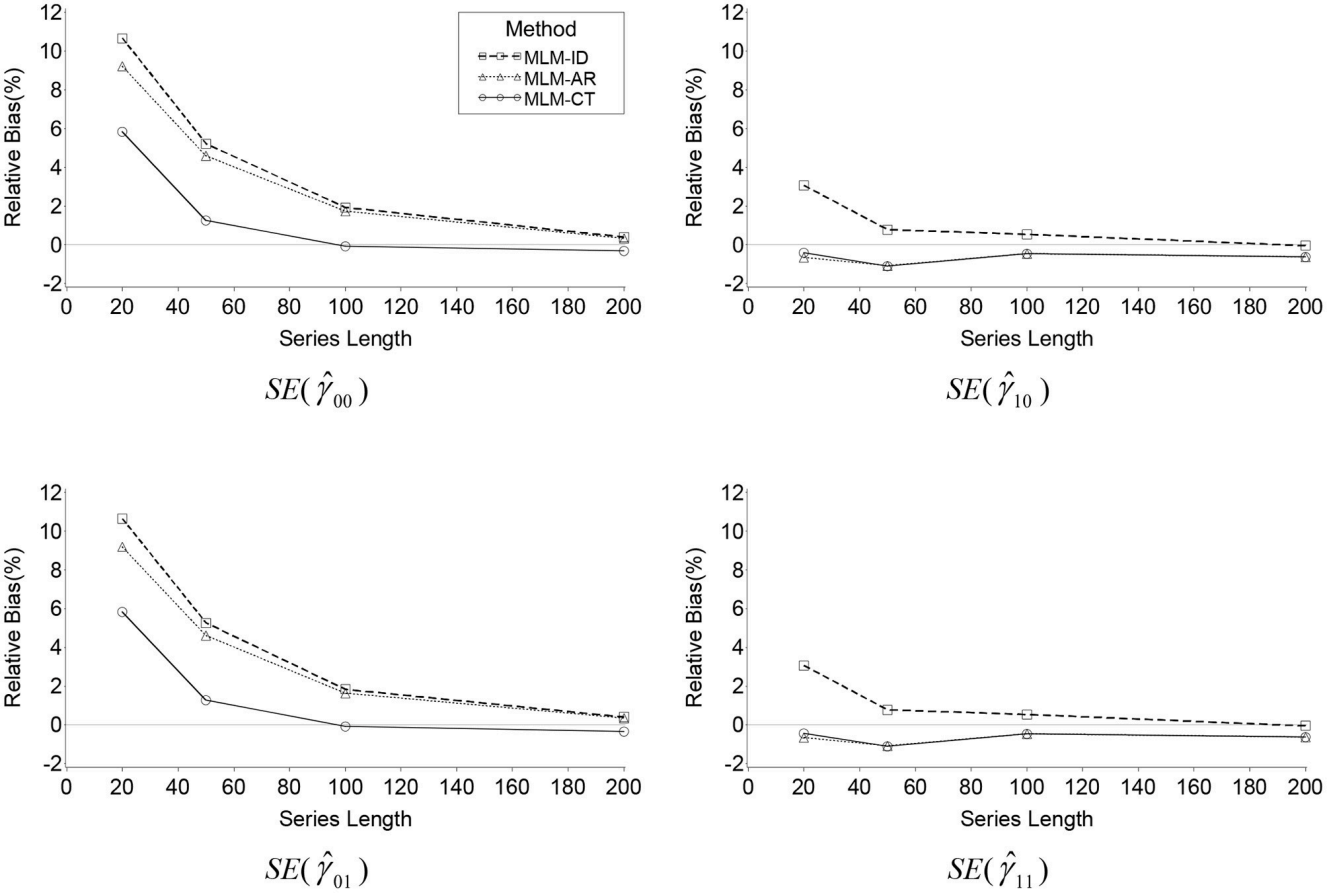
**Line plots of relative bias of the standard error of ${\hat{\gamma }}_{00}$, ${\hat{\gamma }}_{10}$, ${\hat{\gamma }}_{01}$ *and* ${\hat{\gamma }}_{11}$ produced by the three MLMs across series lengths when ρ = 0.6**.

As in the bias of ${\hat{\sigma }}_{u0}^{2}$, relative bias for the standard error of the fixed intercept appeared greater in the ID model than the AR model (η^2^ = 0.04). Bias difference between the two methods was affected by series length (η^2^ = 0.04), autocorrelation (η^2^ = 0.07), and the interaction of the two (η^2^ = 0.05). On the other hand, the MLM-CT showed smaller bias than the AR model (η^2^ = 0.05), where the difference was affected by series length (η^2^ = 0.01), autocorrelation (η^2^ = 0.11), and their interaction (η^2^ = 0.03). The results support that the transformation method is less biased in the estimation of the standard error of ${\hat{\gamma }}_{00}$ than the misspecified models, especially when average autocorrelation is high and the series length is long (see Figure 2).

As seen in Table 3 and Figure 2, the results of the analyses for the standard errors of ${\hat{\gamma }}_{01}$, the fixed effect of the time-invariant covariate *c*_*i*_, were almost identical to those of ${\hat{\gamma }}_{00}$. Overall relative bias of the estimated standard error of ${\hat{\gamma }}_{01}$ was found in the ID model (*RB* = 1.2%) and the AR model (*RB* = 0.9%) but not for the MLM-CT model (*RB* = 0.2%). Bias of the ID model was mainly affected by series length (η^2^ = 0.01), autocorrelation (η^2^ = 0.04), and the interaction of the two (η^2^ = 0.02). Bias of the AR model was affected by series length (η^2^ = 0.01), autocorrelation (η^2^ = 0.03), and the interaction (η^2^ = 0.02). Bias of the MLM-CT model was affected by the interaction of series length by autocorrelation (η^2^ = 0.01).

Relative bias of the standard error of ${\hat{\gamma }}_{10}$, the fixed effect of the time-varying covariate *z*_*ti*_, was statistically significant but their sizes were not practically meaningful. The biases of the three methods were not meaningfully affected by any of the factors considered. The results of the analyses for the standard errors of ${\hat{\gamma }}_{11}$, the fixed effect of the cross-level interaction of *z*_*ti*_ and *c*_*i*_, were near identical to those of ${\hat{\gamma }}_{10}$.

In summary, all three models did not produce biased estimates of the fixed intercept and regression effects. This was also true for estimation of the variance of the random regression effect. Estimation of the variance of the random intercept, however, was severely biased when the average autocorrelation was 0.3 or 0.6, especially for MLM-ID. The MLM-AR was less biased than the MLM-ID in these conditions but the amount of bias was still unsatisfactory. The MLM with Cholesky transformation also produced bias in the estimation of the variance of the random intercept. In fact, the bias of the MLM-CT was higher than MLM-AR when the number of observations within individuals was 20. When the number of observation was large (50 or more), however, the MLM-CT was less biased than the MLM-AR. This was expected before analyzing the data. Because the transformation procedure that actually estimates autoregressive parameters for each individual requires a large number of observations within individuals, the performance of the MLM-CT depends critically on the number of observations. If this is not the case, the first step of the MLM-CT may fail to identify a valid transformation matrix, resulting in poor estimates in the second step. Once enough number of observations are available and analyzed for each individual, however, the MLM-CT produces better estimates than the other misspecified models when residual covariance structure is hetetogeneous. Bias in the estimation of the variance of the random intercept resulted in bias in estimation of the standard error of the fixed intercept and the fixed regression effect of time-invariant covariate. As seen in the results, the MLM-CT may reduce this bias if a large number of observations for each individual (say 50 or more) are available.

## Discussion

In this paper, we discussed issues related to the heterogeneity of residual covariance in multilevel analysis of intensive longitudinal data. We investigated bias in estimation of fixed effects, random components, and standard errors of fixed effects by analyzing large sets of simulated data with heterogeneous autoregressive errors using MLMs with misspecified homogenous ID or AR(1) error structure and the suggested MLM with Cholesky transformation. We found that if homogenous covariance is incorrectly assumed, MLMs produce a highly biased estimate of the variance of random intercepts when the average autocorrelation is high. It is also found that biased estimates of random intercept variance also create biased estimates of the standard error of the fixed intercept and fixed effect of level 2 covariate. For intensive longitudinal data, we saw that application of MLMs to variables transformed by the inverse of Cholesky factor of individual specific residual covariance can be used to reduce the bias.

One obvious result of the present study is that bias of the variance component was found only in the variance of random intercepts but not in the variance of random time-varying effects and the covariance of the two. This effect of misspecification is especially true for MLM with homogenous ID error structure when the average residual autocorrelation is high. This result is in line with findings of the previous studies that investigated the effect of underspecification of homogenous within-person covariance, such as misspecifying homogenous autoregressive error structure as homogenous independent error structure (Ferron et al., 2002; Kwok et al., 2007). As pointed out by Kwok et al., the total covariance matrix of the two-level model is constructed from covariance of between-person random components and covariance of within-person random errors, i.e., **Σ** = **ZGZ**′ + **R**. Because the total covariance **Σ** is primarily determined by a given data set, misspecification of **R** alters the values of elements in **G**, i.e., their relations are compensatory to each other. The results of the present study and other related studies suggest that unmodeled autocorrelation in **R** tends to cause an increased estimate of the variance of random intercepts that helps to explain overall (not first-order) autocorrelation between observations at different time points. Note that **Σ** of the random intercept only model with independent **R** is the same as that of the fixed effects only model (i.e., null **G**) with compound symmetry **R**. Thus, the notable bias of the variance of random intercepts found in MLM with homogenous ID error structure is mainly caused by misspecification of autoregression rather than by misspecification of heterogeneity. This line of reasoning is supported by the fact that the difference of the biases between MLM with homogenous autoregressive error and MLM with Cholesky transformation, an analytical model that takes heterogeneity of residual autoregression into account, is not as big as the differences of the biases between those models and MLM with homogenous independent error.

On the contrary, MLM with homogenous ID errors and MLM with homogenous AR errors showed similar amounts of bias in the standard errors of fixed intercept and fixed effect of time-invariant level-2 covariate when average autocorrelation is high. Because estimates of the standard errors of fixed effects are determined by the estimated covariance matrix of $\hat{\gamma }$, $\hat{C}={\left[X^{\prime }{\hat{\Sigma }}^{-1}X\right]}^{-1}$, the bias of the standard errors of fixed effects depends on the estimated total covariance. Overall it appears that overestimated $\hat{G}$, especially the variance estimate of random intercepts, tend to increase certain elements in $\hat{\Sigma }$ that construct the variances of the estimates of fixed intercept and fixed effect of level-2 covariate. However, difference in $\hat{G}$ does not necessarily create a similar amount of difference in $\hat{\Sigma }$ because $\hat{G}$ and $\hat{R}$ are compensatory to each other. Thus, the greater differences in bias of the standard errors between the two misspecified models and the suggested model with transformation are likely caused by misspecification of heterogeneity, which is not ignorable when average autocorrelation is as high as 0.6.

Another point of the result is that the number of individuals is not an important factor of bias in MLM with ILD. Instead, the number of observations within each individual is a much more important factor of the biases. Thus, if you have intensive longitudinal data with 50 or more repeated observations within individuals and the variable of your interest is highly autocorrelated, the suggested MLM with Cholesky transformation is recommended. If not, there is no practical advantage of using the transformation method and use of MLM with homogenous autoregressive errors has no disadvantage. The transformation method can be applied to EMA data that show different and highly autocorrelated processes with enough number of repeated observations within individuals (e.g., Koval and Kuppens, 2012; Ebner-Priemer et al., 2015).

The idea of transformation to analyze multilevel data has been applied to researchers in other contexts. Moeyaert et al. (2013) standardized a set of single-subject experimental data before employing a three-level regression analysis to synthesize different studies. Lee and Yoo (2014) introduced a Bayesian modeling with Cholesky factor to model random effects covariance matrix for generalized linear mixed models. However, these approaches are not explicitly interested in heterogeneous and autocorrelated error structure.

Several limitations of the present studies are acknowledged. First, because the suggested procedure consists of two steps, it suffers from problems common to any two-step approach. The transformation method requires estimation of autocorrelation or autoregressive parameters of individual time series in the first step and application of the intended MLM on the transformed variables in the second step. As such, a major limitation of the suggested model is a high dependency of the performance in the first step analysis. If poor transformation matrices are obtained in the first step, the final intended MLMs will produce biased estimation of the parameters of interest. For intensive longitudinal data, however, this concern is not critical because performance of the first step analysis gets better as the number of observations within individuals increases. However, for longitudinal data with small to moderate number of observations within individuals, the suggested two-step approaches may produce biased estimation and should not be used. An iterative method alternating the two steps may converge to a better solution.

Another limitation is related to the assessed time intervals between successive measurements. The error covariance structures used in the transformation method in this study assume constant and equally spaced time of measurements. Because ILD are often measured at randomly prompted times, e.g., within-day random assessments of electronic diary, transformation methods introduced in the present study cannot be applied directly to ILD with random time intervals. However, the suggested transformation method should be extended to ILD with random time intervals. In such cases, heterogeneous covariance with autocorrelation that exponentially decreases over random time intervals may be modeled and estimated by individual and then transformation of original variables by multiplying the inverse of the Cholesky factor of the estimated covariance matrix can be applied to get valid estimation of fixed effects and the variance of random effects. Another simulation study can be conducted to evaluate the performance of the transformation method on ILD with random time intervals and heterogeneous autocorrelation.

Intensive longitudinal data are useful to investigate various patterns of intra-individual processes. Although we restricted our discussion on the analysis of ILD to the multilevel models for fixed effects and random components, there are still other possibilities of modeling intra-individual processes using ILD. For example, heterogeneity of autocorrelation can be modeled in MLMs. In this regard, an interesting extension of MLM has been suggested by Rovine and Walls (2006). They showed that the autoregressive parameters can be modeled as random effects in MLM, which allow estimation of individual specific autoregressive parameters. This model has been applied to many EMA studies (e.g., Kuppens et al., 2010; Koval and Kuppens, 2012; Tompson et al., 2012; Bresin, 2014). Hedeker et al. (2008) proposed a multilevel model for EMA data that allows person-specific and time-varying heterogeneous variance. Kapur et al. (2014) extended the model to data with multivariate outcomes by a Bayesian modeling. More recently, multilevel models that simultaneously estimate both heterogeneous autocorrelation and heterogeneous error variance have been applied to ILD with emphasis on Bayesian modeling (Wang et al., 2012; Gasimova et al., 2014; Ebner-Priemer et al., 2015). Jahng et al. (2008) suggested a multilevel random instability model for EMA data where instability is equated as frequent and extreme fluctuations over time and expressed as a function of variance and autocorrelation. Other possibilities for modeling heterogeneous intra-individual process include time-varying regression effects (Fan and Gijbels, 1996; Li et al., 2006), nonlinear multilevel models (Davidian and Giltinan, 1995; Fok and Ramsay, 2006), state space models (Durbin and Koopman, 2001; Ho et al., 2006), and differential equation models (Boker, 2001; Boker and Laurenceau, 2006; Ramsay, 2006).

## Author contributions

SJ was responsible for all aspects of writing, study design, data analysis, and reporting. PW contributed to the literature review and the writing of the manuscript.

### Conflict of interest statement

The authors declare that the research was conducted in the absence of any commercial or financial relationships that could be construed as a potential conflict of interest.

## Footnotes

^1^There is no clear consensus on the number of occasions to define the “intensive” longitudinal data. Walls and Schafer (2006) stated that the traditional longitudinal analysis tends to focus on data with “no more than about ten occasions” in comparison to the analysis of ILD. But, as Bolger and Laurenceau (2013, p. 2) pointed out, specification of a minimum number of measurements for ILD is somewhat arbitrary.

^2^Commercial statistical software such as AUTOREG procedure in SAS can be used for this step. See Appendix for more details.

^3^For unequally spaced observations often encountered in ecological momentary assessment, a continuous time Markov process, $e_{t}=\phi^{h}e_{t-1}+w_{t}$, where ϕ = *e*^−θ^, θ > 0, *w*_*t*_ ~ N(0, $\sigma_{w}^{2}$), *h* ≥ 0, can be used to model **R** and thus the transformation matrix **A**.

^4^The matrix form of the model can be expressed as in Equation 14.

^5^Because all true fixed effects were set to 1, relative biases of the fixed effects are in fact the same as unstandardized biases.

^6^All biases were analyzed using appropriate statistical tests, e.g., *t*-test or ANOVA. In the result section we only reported effect sizes with practical meaning (|RB| ≥ 1.0 % and η^2^ ≥.01) but did not report test statistics and *p*-values because they may distract the major findings of this simulation study with a large number of replications.

## References

[B1] Boker S. M. (2001). Differential structural modeling of intraindividual variability, in New Methods for the Analysis of Change, eds Collins L. Sayer A. (Washington, DC: American Psychological Association), 3–28.

[B2] Boker S. M. Laurenceau J. (2006). Dynamical systems modeling: An application to the regulation of intimacy and disclosure in marriage, in Models for Intensive Longitudinal Data, eds Walls T. A. Schafer J. L. (New York, NY: Oxford University Press), 195–216.

[B3] Bolger N. Laurenceau J. (2013). Intensive Longitudinal Methods: An Introduction to Diary and Experience Sampling Research. New York, NY: The Guilford Press.

[B4] Bresin K. (2014). Five indices of emotion regulation in participants with a history of nonsuicidal self-injury: a daily diary study. Behav. Ther. 45, 56–66. 10.1016/j.beth.2013.09.005 24411115

[B5] Chipman J. S. (1979). Efficiency of least squares estimation of linear trend when residuals are autocorrelated. Econometrica 47, 115–128. 10.2307/1912350

[B6] Cochrane D. Orcutt G. H. (1949). Application of least squares regression to relationships containing autocorrelated error terms. J. Am. Stat. Assoc. 44, 32–61.

[B7] Davidian M. Giltinan D. M. (1995). Nonlinear Models for Repeated Measurement Data. New York, NY: Chapman and Hall.

[B8] Durbin J. Koopman S. J. (2001). Time Series Analysis by State-Space Models. New York, NY: Oxford University Press.

[B9] Ebner-Priemer U. W. Houben M. Santangelo P. Kleindienst N. Tuerlincks F. Oravecz Z. . (2015). Unraveling affective dysregulation in borderline personality disorder: a theoretical model and empirical evidence. J. Abnorm. Psychol. 124, 186–198. 10.1037/abn0000021 25603359

[B10] Fan J. Gijbels I. (1996). Local Polynomial Modeling and Its Applications. London: Chapman and Hall.

[B11] Ferron J. M. Bell B. A. Hess M. R. Rendina-Gobioff G. Hibbard S. T. (2009). Making treatment effect inferences from multiple-baseline data: the utility of multilevel modeling approaches. Behav. Res. Methods 41, 372–384. 10.3758/BRM.41.2.372 19363177

[B12] Ferron J. Dailey R. Yi Q. (2002). Effects of misspecifying the first-level error structure in two-level models of change. Multivariate Behav. Res., 37, 379–403. 10.1207/S15327906MBR3703_4 26751294

[B13] Fok C. C. T. Ramsay J. O. (2006). Fitting curves with periodic and nonperiodic trends and their interactions with intensive longitudinal data, in Models for Intensive Longitudinal Data, eds Walls T. A. Schafer J. L. (New York, NY: Oxford University Press), 109–123.

[B14] Gasimova F. Robitzsch A. Wilhelm O. Hülür G. (2014). A hierarchical Bayesian model with correlated residuals for investigating stability and change in intensive longitudinal data settings. Methodology 10, 126–137. 10.1027/1614-2241/a000083

[B15] Harvey A. C. (1981). The Econometric Analysis of Time Series. New York, NY: John Wiley and Sons.

[B16] Hedeker D. Mermelstein R. J. Demirtas H. (2008). An application of a mixed-effect location scale model for analysis of ecological momentary assessment (EMA) data. Biometrics 64, 627–634. 10.1111/j.1541-0420.2007.00924.x 17970819 PMC2424261

[B17] Hill C. L. Updegraff J. A. (2012). Mindfulness and its relationship to emotional regulation. Emotion 12, 81–90. 10.1037/a0026355 22148996

[B18] Ho M. R. Shumway R. Ombao H. (2006). The state-space approach to modeling dynamic processes, in Models for Intensive Longitudinal Data, eds Walls T. A. Schafer J. L. (New York, NY: Oxford University Press), 148–175.

[B19] Hufford M. R. Shiffman S. Paty J. Stone A. A. (2001). Ecological momentary assessment: real-world, real-time measurement of patient experience, in Progress in Ambulatory Assessment: Computer-Assisted Psychological and Psych Physiological Methods in Monitoring and Field Studies, eds Fahrenberg J. Myrtek M. (Kirkland, WA: Hogrefe and Huber), 69–92.

[B20] Jacqmin-Gadda H. Sibillot S. Proust C. Molina J. Thiébaut R. (2007). Robustness of the linear mixed model to misspecified error distribution. Comput. Stat. Data Analysis 51, 5142–5154. 10.1016/j.csda.2006.05.021

[B21] Jahng S. (2008). Multilevel Models for Intensive Longitudinal Data with Heterogeneous Error Structure: Covariance Transformation and Random Variance Models. Dissertation, Columbia, MO, University of Missouri.

[B22] Jahng S. Wood P. K. Trull T. J. (2008). Analysis of affective instability in ecological momentary assessment: Indices using successive difference and group comparison via multilevel modeling. Psychol. Methods 13, 354–375. 10.1037/a0014173 19071999

[B23] Judge G. G. Griffiths W. E. Hill R. C. Lütkepohl H. Lee T. C. (1985). The Theory and Practice of Econometrics, 2nd Edn. New York, NY: John Wiley and Sons.

[B24] Kapur K. Li X. Blood E. A. Hedeker D. (2014). Bayesian mixed-effects location and scale models for multivariate longitudinal outcomes: an application to ecological momentary assessment data. Stat. Med. 34, 630–651. 10.1002/sim.6345 25409923 PMC4768818

[B25] Koreisha S. G. Fang Y. (2001). Generalized least squares with misspecified serial correlation structures. J. R. Stat. Soc. Ser. B 63, 515–531. 10.1111/1467-9868.00296

[B26] Koval P. Kuppens P. (2012). Changing emotion dynamics: individual differences in the effect of anticipatory social stress on emotional inertia. Emotion 12, 256–267. 10.1037/a0024756 21787072

[B27] Kuppens P. Allen N. B. Sheeber L. (2010). Emotional inertia and psychological maladjustment. Psychol. Sci. 21, 984–991. 10.1177/0956797610372634 20501521 PMC2901421

[B28] Kwok O. West S. G. Green S. B. (2007). The impact of misspecifying the within-subject covariance structure in multiwave longitudinal multilevel models: a monte carlo study. Multivariate Behav. Res. 42, 557–592. 10.1080/00273170701540537

[B29] Lange N. Laird N. (1989). The effect of covariance structure on variance estimation in balanced growth-curve models with random parameters. J. Am. Stat. Assoc. 84, 241–247 10.1080/01621459.1989.10478761

[B30] Lee K. Yoo J. K. (2014). Bayesian Cholesky factor models in random effects covariance matrix for generalized linear mixed models. Comput. Stat. Data Analysis 80, 111–116. 10.1016/j.csda.2014.06.016

[B31] Li R. Root T. L. Shiffman S. (2006). A local linear estimation procedure for functional multilevel modeling, in Models for Intensive Longitudinal Data, eds Walls T. A. Schafer J. L. (New York, NY: Oxford University Press), 63–83.

[B32] Maeshiro A. (1980). Small sample properties of estimators of distributed lag models. Int. Econ. Rev. 21, 721–733. 10.2307/2526364

[B33] Moeyaert M. Ugille M. Ferron J. Beretvas S. N. Van den Noortgate W. (2013). Three-level analysis of standardized single-case experimental data: empirical validation. Multivariate Behav. Res. 48, 719–748. 10.1080/00273171.2013.816621 26741060

[B34] Moeyaert M. Ugille M. Ferron J. Beretvas S. N. Van den Noortgate W. (2016). The misspecification of the covariance structures in multilevel models for single-case data: a monte carlo simulation study. J. Exp. Educ. 84, 473–509. 10.1080/00220973.2015.1065216

[B35] Nezlek J. B. (2012). Multilevel modeling analyses of diary-style data, in Handbook of Research Methods for Studying Daily Life, eds Mehl M. R. Conner T. S. (New York, NY: The Guilford Press), 357–383.

[B36] Park R. E. Mitchell B. M. (1980). Estimating the autocorrelated error model with trended data. J. Econom. 13, 185–201. 10.1016/0304-4076(80)90014-7

[B37] Ramsay J. O. (2006). The control of behavioral input/output systems, in Models for Intensive Longitudinal Data, eds Walls T. A. Schafer J. L. (New York, NY: Oxford University Press), 176–194.

[B38] Röcke C. Li S.-C. Smith J. (2009). Intraindividual variability in positive and negative affect over 45 days: do older adults fluctuate less than young adults? Psychol. Aging 24, 863–878. 10.1037/a0016276 20025402

[B39] Rovine M. J. Walls T. A. (2006). Multilevel autoregressive modeling of differences in the stability of a process, in Models for Intensive Longitudinal Data, eds Walls T. A. Schafer J. L. (New York, NY: Oxford University Press), 124–147.

[B40] SAS Institute Inc (2013). SAS/ETS® 12.3 User's Guide. Cary, NC: SAS Institute Inc.

[B41] Schwartz J. E. Stone A. A. (2007). The analysis of real-time momentary data: A practical guide, in The Science of Real-Time Data Capture: Self-Reports in Health Research, eds Stone A. Shiffman S. Arienza A. Nebeling L. (New York, NY: Oxford University Press), 76–113.

[B42] Shumway R. H. Stoffer D. S. (2011). Time Series Analysis and Its Applications, 3rd Edn. New York, NY: Springer.

[B43] Stone A. A. Shiffman S. (1994). Ecological momentary assessment in behavioral medicine. Ann. Behav. Med. 16, 199–202.

[B44] Tompson R. J. Mata J. Jaeggi S. M. Buschkuehl M. Jonides J. Gotlib I. H. (2012). The everyday emotional experience of adults with major depressive disorder: examining emotional instability, inertia, and reactivity. J. Abnorm. Psychol. 121, 819–829. 10.1037/a0027978 22708886 PMC3624976

[B45] Walls T. A. Jung H. Schafer J. L. (2006). Multilevel models for intensive longitudinal data, in Models for Intensive Longitudinal Data, eds Walls T. A. Schafer J. L. (New York, NY: Oxford University Press), 3–37.

[B46] Walls T. A. Schafer J. L. (Eds.). (2006). Models for Intensive Longitudinal Data. New York, NY: Oxford University Press.

[B47] Wang L. P. Hamaker E. Bergeman C. S. (2012). Investigating inter-individual differneces in short-term intra-individ ual variability. Psychol. Methods 17, 567–581. 10.1037/a0029317 22924600 PMC3684184

[B48] Watson G. S. (1955). Serial correlation in regression analysis. I. Biometrika 42, 327–341. 10.1093/biomet/42.3-4.327

