# Supplementary material for: Multilevel Models for Intensive Longitudinal Data with Heterogeneous Autoregressive Errors: The Effect of Misspecification and Correction with Cholesky Transformation
Source: Front Psychol. 2017 Feb 24;8:262. doi: 10.3389/fpsyg.2017.00262 (PMC5323419; doi:10.3389/fpsyg.2017.00262)
Supplement: Supplementary file 1 [file DataSheet1.pdf]

Appendix

Following SAS code is an illustration of the multilevel modeling with Cholesky transformation. The first part is written to simulate a multilevel data with heterogeneous autoregressive errors. The following two-level linear mixed model was used to generate data:

$$y_{ti} = \gamma_{00} + \gamma_{10} z_{ti} + \gamma_{01} c_i + \gamma_{11} c_i z_{ti} + u_{0i} + u_{1i} z_{ti} + e_{ti} \,,$$

$$\begin{pmatrix} u_{0i} \\ u_{1i} \end{pmatrix} \sim N\left(\begin{bmatrix} 0 \\ 0 \end{bmatrix}, \begin{bmatrix} \sigma^2_{u0} & \\ \sigma_{u0u1} & \sigma^2_{u1} \end{bmatrix} \right),$$

$$e_{ti} = \rho_i e_{(t-1)i} + w_{ti}, w_{ti} \sim N(0,1)\,.$$

Parameters were set to as follows: γ_{00} = 1.0, γ_{10} = 0.3, γ_{01} = 0.2, γ_{11} =-0.1, σ^2_{u0} = 0.5, σ^2_{u1} = 0.5, σ_{u0u1} = 0.15 (i.e., r_{u0u1} = .3), and $\rho_i \sim U(0, 0.6)$. The time-varying covariate z_{ti} and the time-invariant covariate c_i were generated from the standard normal distribution. The number of individuals and the number of occasions were both 50. The numbers in parenthesis are random number seeds.

```
/*SAMPLE DATA GENERATION*/
*GENERATING A RANDOM-EFFECT VECTOR BETAS;
      PROC IML;
            var_a=.5;
            var_b=.5;
            corr_ab=.3;
            cov_ab=corr_ab*sqrt(var_a)*sqrt(var_b);
            g=(var_a||cov_ab)//(cov_ab||var_b);
            n=50;
            call randseed(123);
            u=RandNormal(n,{0,0},g);
            CREATE u FROM u [colname={"u0i" "u1i"}];
            APPEND FROM u;
      QUIT;
      DATA betas;
            SET u;
            gamma00=1;*intercept;
            gamma10=.3;*within-individual covariate effect;
            gamma01=.2;*between-individual covariate effect;
            gamma11=-.1;*cross-level interaction effect;
            c=rannor(234);*between-individual covariate;
            b0i=gamma00 + gamma01*c + u0i;
            b1i=gamma10 + gamma11*c + u1i;
      RUN;
*GENERATING HETEROGENEOUS AR(1) PARAMETERS;
      DATA sigma;
            DO id=1 TO 50;
            rho=.6*ranuni(345);
            OUTPUT;
            END;
      RUN;
```

```
* GENERATING FULL DATA WITH REPEATED OCCASIONS;
      DATA sample;
            MERGE betas sigma;
            e=rannor(456);
            DO i=-20 TO 50;
            z=rannor(1234);
            zc=z*c;
            e=rho*e+rannor(5678);
            y=b0i+b1i*z+e;
            int=1;
            IF i>0 THEN OUTPUT;
            END;
      RUN;
```

The second part is written to analyze the generated data using multilevel model with homogenous independent error structure (MLM-ID), multilevel model with homogenous AR(1) error structure (MLM-AR), and multilevel model with Cholesky transformation (MLM-CT), respectively.

```
/*MULTILEVEL ANALYSES OF THE SAMPLE DATA*/
*MLM WITH HOMOGENOUS ID ERROR STRUCTURE;
      PROC MIXED data=sample;
            CLASS id;
            MODEL y =z c zc/solution;
            RANDOM int z/sub=id type=un;
      RUN;
*MLM WITH HOMOGENOUS AR(1) ERROR STRUCTURE;
      PROC MIXED data=sample;
            CLASS id;
            MODEL y =z c zc/solution;
            RANDOM int z/sub=id type=un;
            REPEATED /sub=id type=ar(1);
      RUN;
*MLM WITH HETEROGENEOUS AR(1) ERROR STRUCTURE USING CHOLESKY
TRANSFORMATION;
      PROC AUTOREG data=sample noprint;
            BY id;
            MODEL y=z/nlag=1 method=ml;
            OUTPUT out=trans constant=t_int
                  transform=y z c zc;
      RUN;
      PROC MIXED data=trans;
            CLASS id;
            MODEL y=t_int z c zc/noint solution;
            RANDOM t_int z/sub=id type=un;
      RUN;
```

Note that, in the first step of the transformation method using the PROC AUTOREG procedure, all variables that will be modeled in the second step including time-invariant covariate c_i and the cross-level interaction term $c_i z_{ti}$ should be transformed as specified in the "`transform=`"

option of the "OUTPUT" statement. This is also true for the intercept as specified in the "constant=" option of the same statement. This transformed intercept, instead of untransformed intercept of 1's implicitly used by default, should be used in the PROC MIXED procedure of the second step as specified by adding the transformed intercept "t_int" as an explaining variable with "noint" option in the "MODEL" statement. The results of the three analyses were presented in the following table.

	MLM-ID			MLM-AR			MLM-CT		
Parameter	*Estimate*	*SE*	*p*	*Estimate*	*SE*	*p*	*Estimate*	*SE*	*p*
Fixed Effects									
γ_{00}	1.137	0.100	<.0001	1.133	0.100	<.0001	1.137	0.099	<.0001
γ_{10}	0.386	0.086	<.0001	0.379	0.084	<.0001	0.378	0.083	<.0001
γ_{01}	0.272	0.114	0.017	0.271	0.115	0.018	0.274	0.114	0.016
γ_{11}	-0.208	0.098	0.034	-0.224	0.096	0.020	-0.220	0.096	0.022
Random Components									
σ_{u0}^2	0.468			0.451			0.441		
σ_{u0u1}	0.215			0.206			0.196		
σ_{u1}^2	0.338			0.327			0.325		
ρ				0.303					
σ_e^2	1.160			1.180					
σ_w^2							1.018		
